# Total knee arthroplasty with simultaneous tibial shaft osteotomy in patient with multiple hereditary osteochondromas and multiaxial limb deformity – a case report

**DOI:** 10.1186/s12891-020-03245-x

**Published:** 2020-04-13

**Authors:** Dariusz Grzelecki, Jan Szneider, Dariusz Marczak, Jacek Kowalczewski

**Affiliations:** 1 Department of Orthopaedics and Rheumoortopaedics, Professor Adam Gruca Teaching Hospital, Centre of Postgraduate Medical Education, Konarskiego 13, 05-400 Otwock, Poland; 2 Department of Traumatology and Orthopaedic Surgery, Professor Adam Gruca Teaching Hospital, Centre of Postgraduate Medical Education, Konarskiego 13, 05-400 Otwock, Poland

**Keywords:** Hereditary multiple exostoses, Total knee arthroplasty, Tibial shaft osteotomy

## Abstract

**Background:**

Hereditary multiple osteochondromas (hereditary multiple exostoses, HME) is a rare genetic disease characterized by the development of benign osteocartilaginous tumors that may cause severe limb deformities and early onset osteoarthritis. Total knee arthroplasty (TKA) is the method of choice for the treatment of advanced gonarthrosis, however the surgical management with coexisting severe axial limb deformity remains unclear.

**Case presentation:**

65-year-old man with HME and extra-articular multi-axial limb deformity was admitted to the orthopedic department due to chronic knee pain and limited range of motion caused by secondary osteoarthritis. Regarding to the clinical and radiological examinations, after preoperative planning he was qualified to a one-stage TKA combined with tibial shaft osteotomy (TSO). In a one year follow-up full bone union was confirmed with no signs of implant loosening or prosthesis displacement. Patient was very satisfied, did not report any joint pain and has sufficient range of motion without knee instability.

**Conclusion:**

The improvement of mechanical axis during TKA is a crucial factor for achieve operative success and long implant survival. Despite the higher risk of complication rate in comparison to two-stage treatment, one-stage TKA with simultaneous TSO should be a considerable method for patients with osteoarthritis and multiaxial limb deformities. This method can give a good clinical and functional outcomes, however should be performed subsequently to careful preoperative planning and proper patient qualification.

## Background

Hereditary multiple osteochondromas (hereditary multiple exostoses, HME) is an autosomal dominant genetic disorder belongs to skeletal dysplasia group characterized by appearance of benign osteocartilaginous excrescences. It is known that HME is associated with mutation of *ext* family tumor suppressor genes linked to chromosomes 8, 11 and 19 [1]. The most of the patients have a positive family history, but 10–20% of cases are a result of spontaneous mutation [2]. The estimated incidence of the disease is 1:50000 (0.002% of general population) with the men to women ratio 1.5:1 [3].

Exostoses usually appear bilaterally and involve long bones but also can be found in scapula and ribs. Approximately 2.7% of affected individuals are at risk of development of malignant sarcoma transformation [3]. Lower limb deformities with the prevalence to distal femur and proximal tibia are common for HME (involvement ranging from 70 to 98%) and may cause chronic pain and early onset osteoarthritis [4].

Total knee arthroplasty (TKA) is the method of choice for the treatment of advanced gonarthrosis. Concomitant extra-articular deformities make this procedure more challenging and require careful preoperative planning, application of additional revision components, adequate soft tissue balancing and/or limb correction osteotomies [5].

We present a case report of a patient with HME with multi-axial lower limb deformity who has been successfully treated by a one-stage TKA with simultaneous 1/3 proximal tibial shaft osteotomy (TSO) with the stable fixation of the osteotomy by a well matched prosthesis stem, without the application of additional fixation. No similar cases treated in a way proposed by the authors have been found in the medical literature.

## Case presentation

A 65-year-old man (75 kg, 170 cm, BMI = 25,95 kg/m^2^) with HME and extra-articular multi-axial lower limb deformity was admitted to the orthopedic department due to a severe continual right knee pain caused by secondary osteoarthritis (Kellgren-Lawrence grade IV). He was operated on several times in the past due to a chronic pain caused by nerves compression by a large exostoses, which did not revealed malignant transformation in histopathological examinations. Although, he did not have any traumatic episode before but reported gradual knee distortion. Physical examination revealed highly restricted range of motion with 30^○^ flexion contracture, 110^○^ of flexion range and 30^○^ valgus deformation with medial and antero-posterior knee instability. He scored 5 points in a Lovett’s muscle strength scale. No neurovascular disorders were found. Additionally, patient reached 2 points in clinical and 40 points in functional Knee Society Score (KSS) evaluation.

**Fig. 1:**
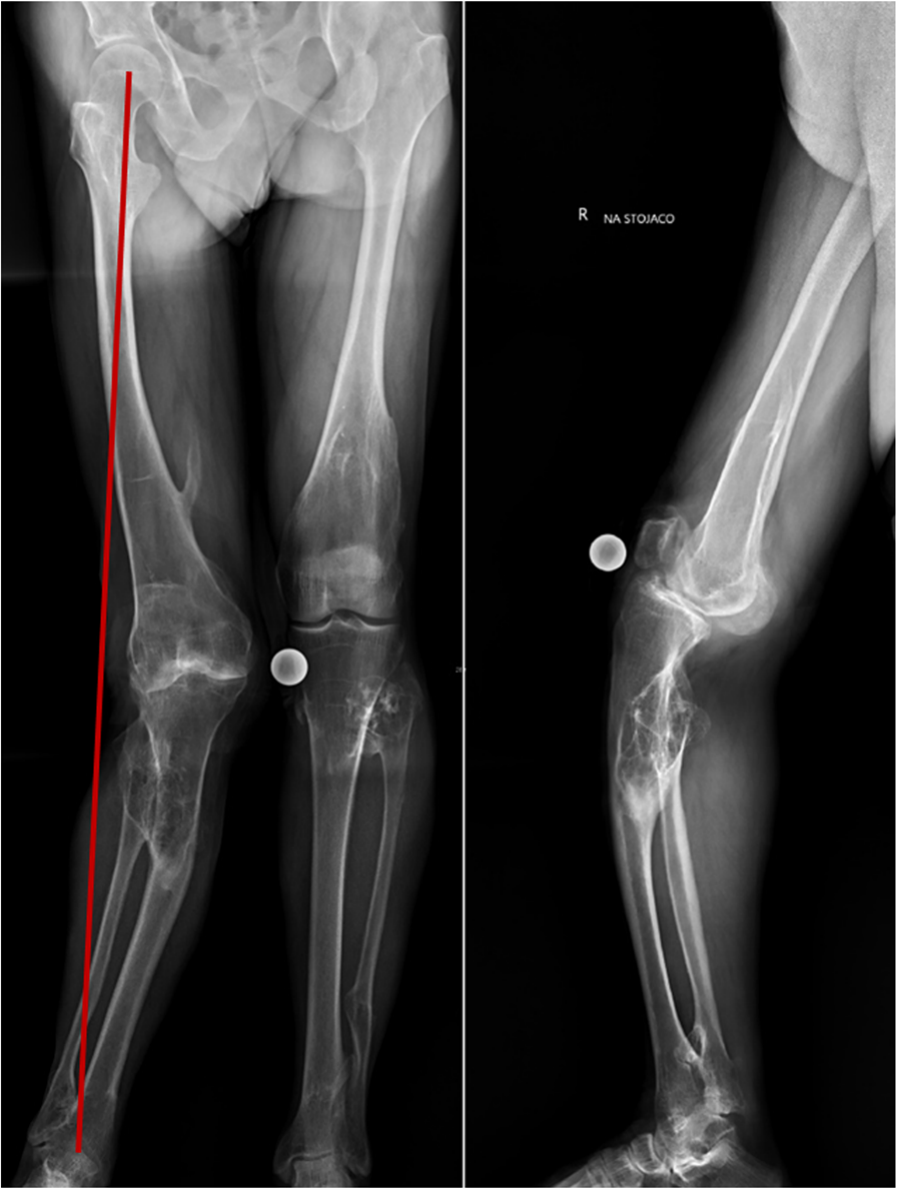
65-year-old man with HME and extra-articular deformity. Long standing x-rays AP and lateral show knee osteoarthritis with mechanical axis deviation and proximal tibio-fibular adhesion

**Fig. 2:**
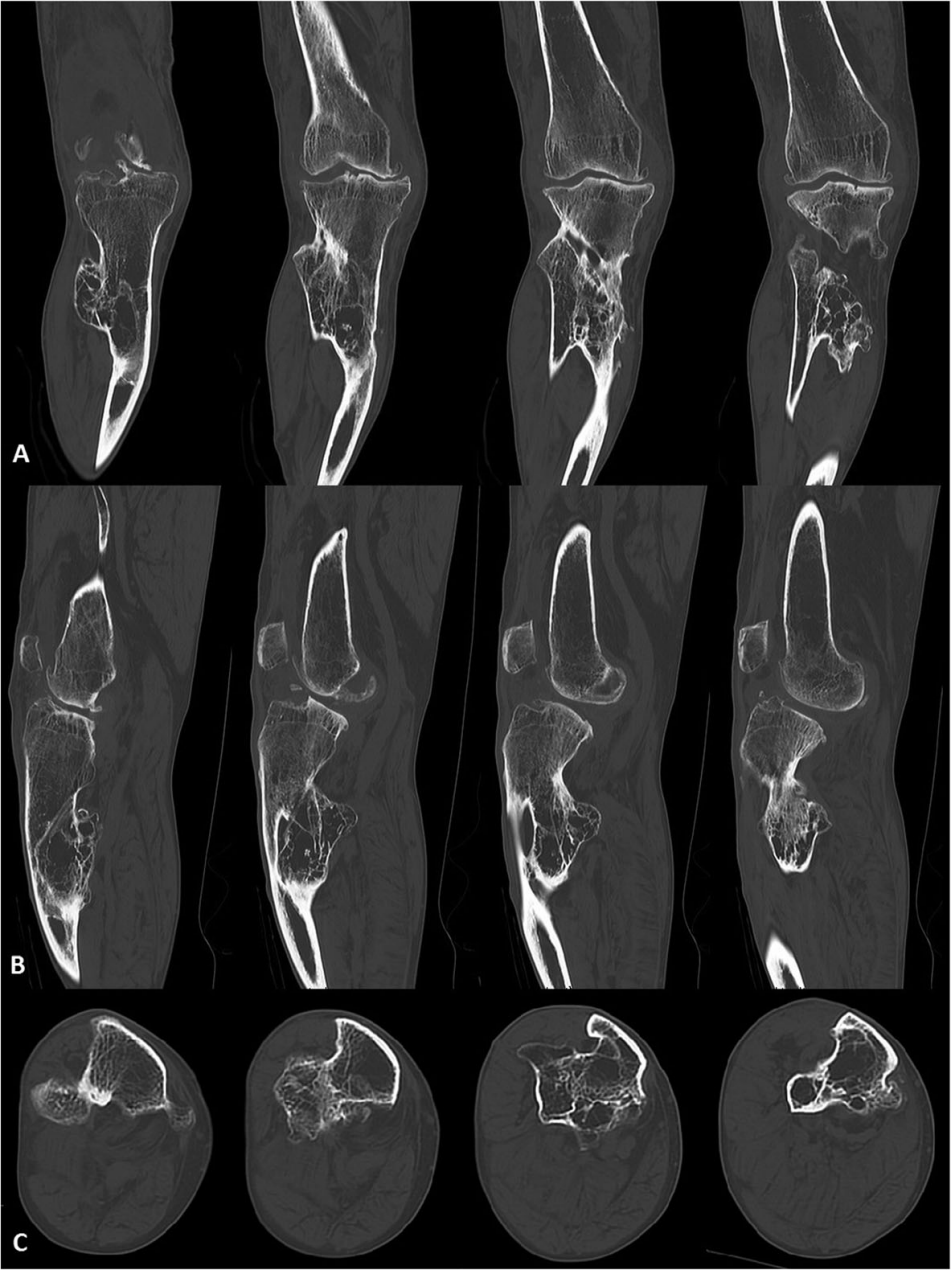
CT scans in **a** – coronal, **b** – sagittal, **c** – horizontal planes of the right knee

**Fig. 3:**
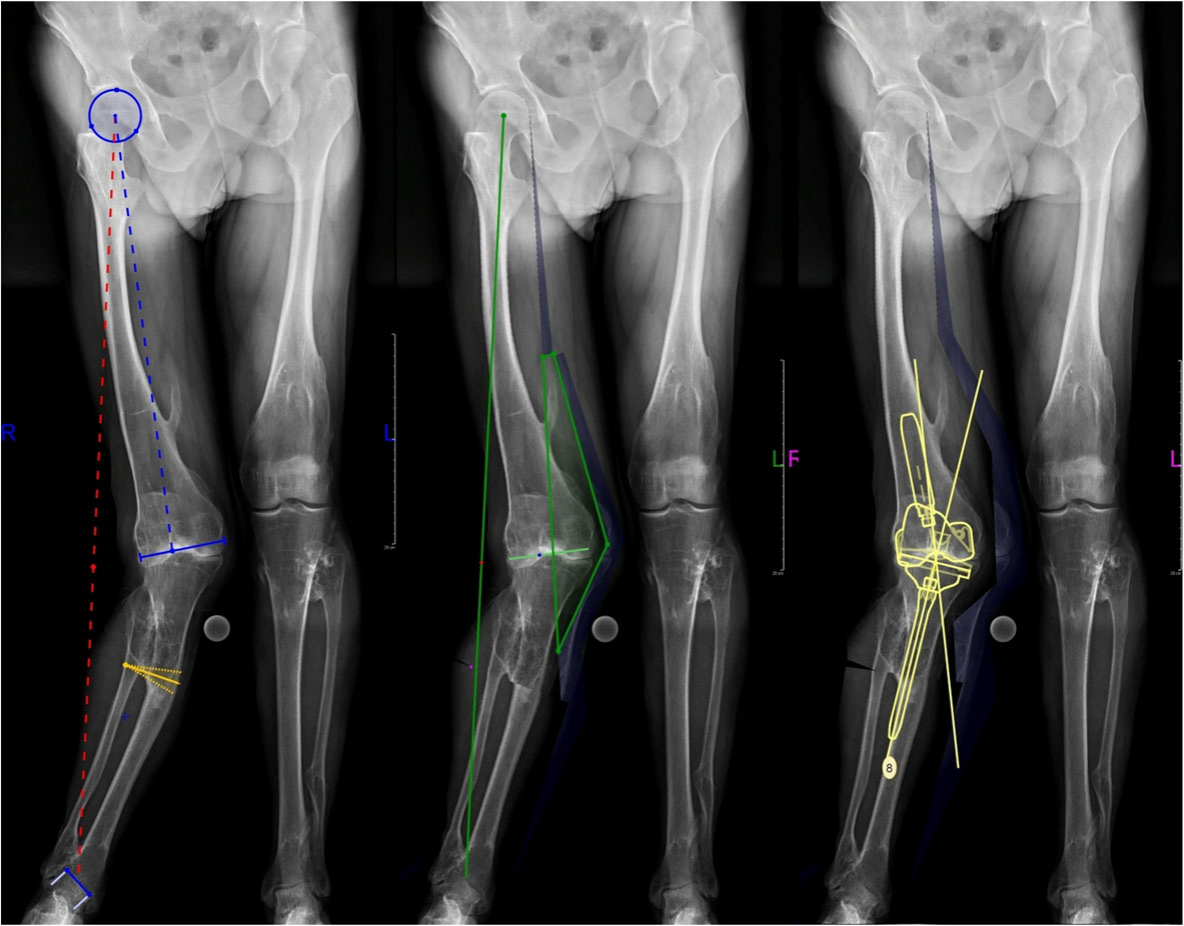
Preoperative planning in OrthoView™. Osteotomy protocol and prosthesis matching

A long standing x-ray (AP and lateral view) and computed tomography (CT) were done. These examinations showed bilateral valgus femoral necks, 20^○^ coronal and 40^○^ sagittal tibial plane deviation, 15^○^ valgus knee deformity with anterior subluxation, proximal tibio-fibular adhesion and 20^○^ ankles deformations (Figs. 1 and 2). Regarding to clinical and radiological assessment, patient was qualified to one-stage TKA combined with TSO. Preoperative osteotomy planning and digital prosthesis templating were done with the use of OrthoView™ software (Fig. 3). Due to relatively low risk of development of malignant sarcoma transformation, negative histopathological findings in the past (after exostoses resections), no changes in preoperative x-rays in relation to x-rays made two years before the surgery, a bone scan to exclude malignant lesions was not performed.

A midline skin incision elongated to the level of tibia’s deformation was done. Closed-wedge osteotomy was set in preoperatively planned CORA point (Center of Rotation and Angulation) at the level of tibia deformation, 8 cm below plateau. The osteotomy was closed without cutting of the fibula, after osteophytes resection and partial release of lower part of tibio-fibular adhesion. Subsequently, parapatellar approach was made and the bone cuts were performed. Femur first technique was applied. After the femoral bone cuts, tibial subsequent intramedullary reamers were used. The osteotomy was temporary stabilized on final reamer in proper rotation and alignment. Following the surgical technique, proximal tibial resection guide was slide over the top of the reamer and the cut was done. To improve implant stability semi-constrained condylar knee prosthesis was used (Triathlon TS, Stryker, Mahwah, USA; femoral implant No.5 with 100 mm stem and 20 mm diameter, tibial implant No.4 with 150 mm stem and 10 mm diameter and poly insert 9 mm). In relation to preoperative CT planning, 8 mm tibial offset at 3 o’clock was confirmed intraoperatively and prepared by a bushing guide. Hybrid cementation technique with cemented tibial baseplate and femoral component, without stems cementation was used. Bone fragments at the osteotomy level in proper axis and rotation were immobilized intramedullary by matched press-fit cementless tibial stem without the additional fixation materials. At the end of the surgery, full extension with 120^○^ of flexion range, proper patellar traction and joint stability were achieved.

**Fig. 4:**
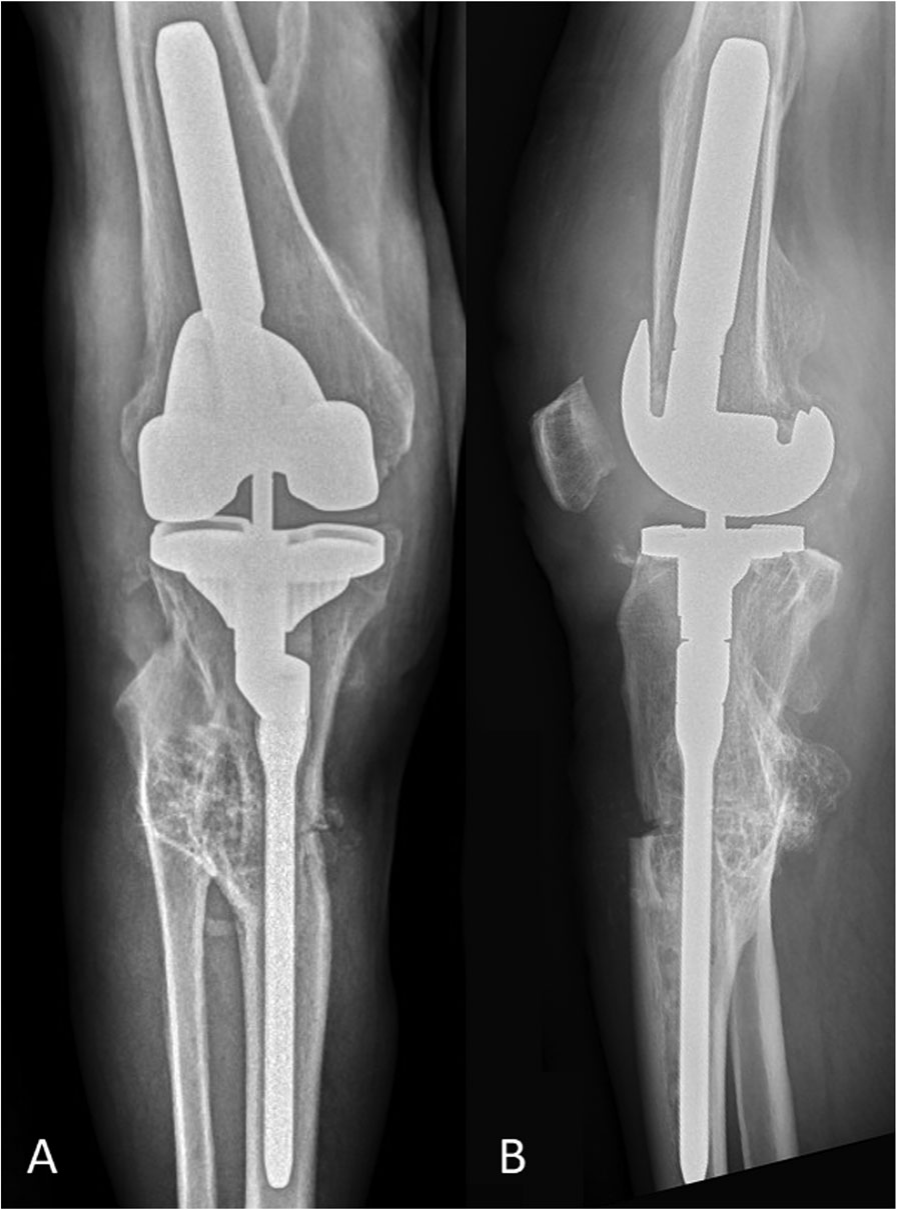
X-ray views (**a**) AP and (**b**) lateral 10 days after TKA

Full weight-bearing with flexion up to 90^○^ was allowed immediately. To avoid the rotation moves patient was equipped with a long knee stabilizer. Walking with the assurance of 2 crutches with above postoperative care strategy was recommended for 6 weeks. Long standing x-ray in two projections was performed 10 days after the surgery (Fig. 4).

The first follow-up visit was six weeks after the surgery. Long standing x-rays in two projections revealed no signs of implant loosening or bone fragments malposition. Patient had range of motion from 0 to 80^○^ with the joint stability in all planes. He did not report the knee pain and gait problems.

**Fig. 5:**
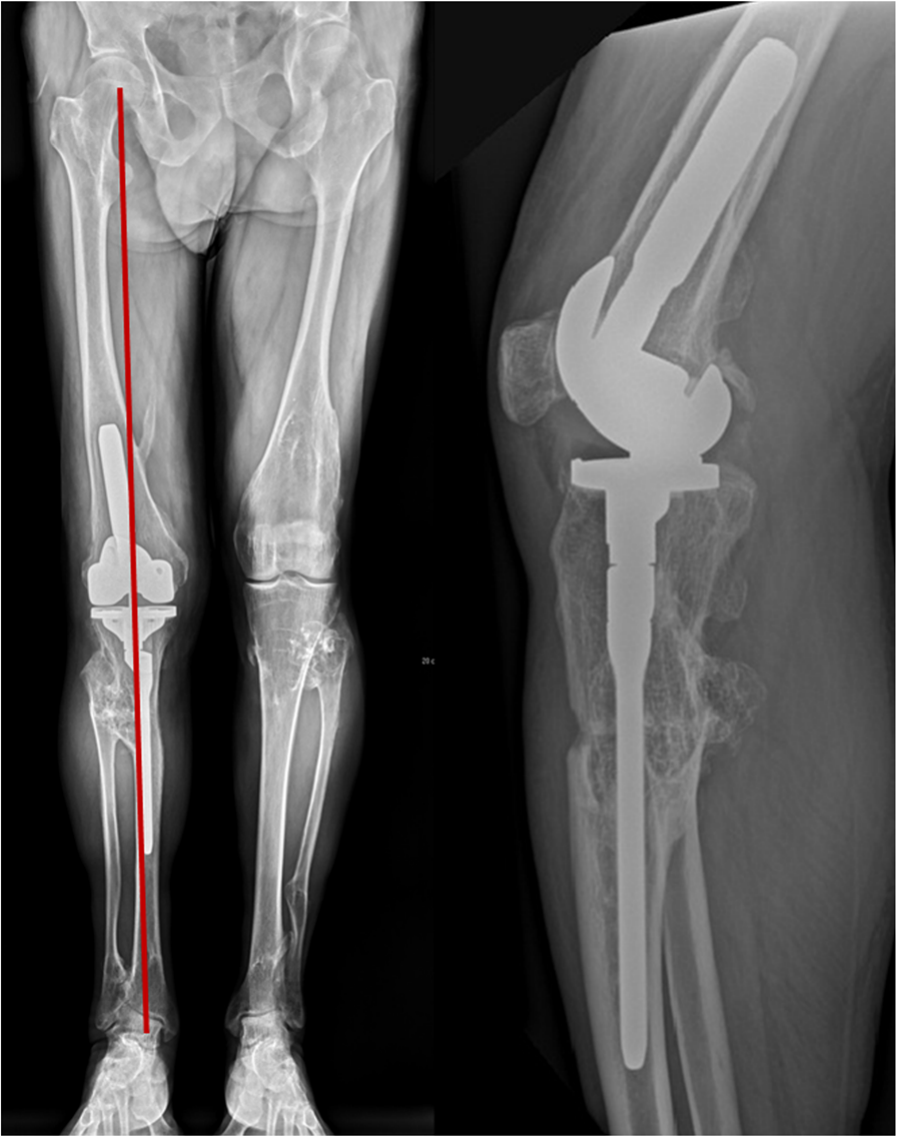
Long standing x-ray AP and lateral one year after TKA

One year after surgery, bone union was confirmed and the patient was clinically asymptomatic (Fig. 5). KSS evaluation revealed 80 points in clinical and 75 points in functional part that showed significant increase of his efficiency. He had full stability of the operated knee, painless range of motion from 0 to 110^○^ and could perform the most of daily activities. He was very satisfied with the surgical outcome and significantly improved the quality of life.

## Discussion

The malunion of lower extremity fractures, failure of corrective osteotomies and genetic or metabolic bone disorders are recognized as the most frequent causes of alignment deviations [6]. The improvement of mechanical axis, proper mechanical load and stable fixation are the crucial factors to achieve operative success [7, 8]. It is estimated that up to 12% of patients with extra-articular deformities can be affected by knee osteoarthritis that makes this problem common for orthopedic surgeons [9]. Only a few cases of TKA in patients with skeletal dysplasia such as HME have been reported in medical literature. The reported cases indicate a good clinical and functional outcomes in short-term follow-up, however these results were strongly related to joint destruction, severity of deformity, proper implant selection and soft tissue balancing [10, 11].

Extra-articular deformities of the femur or tibia implementing the additional difficulty to TKA. In agreement with Mesfin et al. we emphasizing the importance of precise preoperative planning to determine whether the deformation can be correct intra-articular by implant match and soft tissue balancing or extra-articular by the osteotomy [10]. In the most of cases semi-constrained or constrained knee implants with augments and offsets combined with asymmetric resection can be successfully performed with good clinical and functional outcomes [12]. In case of severe extra-articular deformities located distally to the joint line to restore the limb axis and mechanical load the additional osteotomy may be required for improve the knee function and longer prosthesis survival. Current options assume the possibility of one- or two-stage surgery.

One-stage TKA combined with tibial or femoral osteotomy are technically more demanding and challenging procedure which should be performed by experienced surgeons. This procedure seems to be more risky regarding to potential complications such as instability, nonunion, limited range of motion or infections [13]. In case of two-stage procedures involving osteotomy and subsequent TKA after bone union, we agree with Radke et al. that this method is not acceptable for patients [14].

Recent studies have described the cases of TKA combined with high tibial osteotomy (HTO) [14–16] or femoral shaft osteotomy [17] however, the reports which evaluate the results of single-stage TKA with TSO fixed intramedullary by a prosthesis stem have not been found in medical literature. It has been confirmed that the one-stage HTO combined with TKA gives comparable results in KSS to TKA performed as a second-stage after the osteotomy without patella-related and bone union problems [14]. Madelaine et al. reported good clinical results for varus knees with the complication frequency 46.7% (4 tibial plateau fractures, 2 non unions and one deep infection per 15 procedures) and 86,7% implant survival at 22 months [15]. Respectively, Veltman et al. received lower rate (18.2%) of complication (2 cases of deep infection per 11 TKA with HTO) and explained this phenomenon by more vulnerable tissues covering tibia [16]. Furthermore, it was confirmed that rigid fixation of the osteotomy is essential to achieve bone union and good postoperative result [15]. Additionally, some of the authors recommend the use of combination of fixation materials and bone grafts to prevent implant migration, early loosening, progressive angular deviation and to improve bone union [18]. Although, bone grafts were considered before the surgery, in this case but due to intraoperatively confirmed stable immobilization of the bone fragments their use was abandoned.

In presented case report, hybrid cementation technique of tibial implant was done – cemented baseplate and press-fit stem. We believe that implicated method allows to avoid potential bone union problems at the level of osteotomy with sufficient bone fragments and implant fixation. Despite to our results, radiostereometrical studies have shown significant differences between hybrid cementation technique compared to full tibial implant cementation in case of prosthesis migration (rotational *p* = 0.01; subsided *p* = 0.02), however no differences in functional and clinical outcomes were found [19]. Comparable results were found by Heesterbeek et al. who have confirmed in their study no differences between all-cement and hybrid fixation in equal stability and clinical outcomes at 24 months after revision TKA [20].

One-stage TKA with TSO should be a considerable treatment method for patients with knee osteoarthritis with coexisting extra-articular, multi-axial limb deformities. It is technically demanding procedure recommended for experienced surgeons who have to consider the higher risk of failure. Precise preoperative planning, matched long stemmed prosthesis, offsets, adequate soft tissue balance and rigid fixation of the osteotomy are important factors to achieve good clinical and functional results. We believe that proposed method, with the intramedullary fixation by a well matched prosthesis stem, can be successfully applied in a specific group of patients, although further research on a larger group of patients are needed to evaluate the efficacy and safety of simultaneous TKA with TSO.

## Data Availability

Not applicable to this article, no datasets were analyzed.

## Abbreviations

CT: Computed Tomography
CORA: Center of Rotation and Angulation
HME: Hereditary Multiple Exostoses
KSS: Knee Society Score
HTO: High Tibial Osteotomy
TKA: Total Knee Arthroplasty
TSO: Tibial Shaft Osteotomy
